# Older adults coping with critical life events - results of the revised demoralization scale in a representative sample of older adulthood

**DOI:** 10.3389/fpsyt.2024.1389021

**Published:** 2024-05-10

**Authors:** Markus Ramm, Johanna Jedamzik, Philipp Lenz, Lara Jürgens, Gereon Heuft, Rupert Conrad

**Affiliations:** ^1^ Department of Psychosomatic Medicine and Psychotherapy, University Hospital Münster, Münster, Germany; ^2^ West German Cancer Center, University Hospital Münster, Münster, Germany; ^3^ Institute of Palliative Care, University Hospital Münster, Münster, Germany

**Keywords:** depression, older adulthood, demoralization, suicidality, cancer, demoralization scale

## Abstract

**Background:**

High suicide rates in older adults are a relevant public health concern. Social isolation or widowhood as well as physical decline play a crucial role for suicidality in older adulthood. Previous evidence suggested that demoralization is an important risk factor for suicide. Whether demoralization is a relevant phenomenon in older adulthood which possibly could account for high suicide rates remains unclear.

**Methods:**

Demoralization Scale II (DS-II) scores assessed in a survey of the German general population were investigated with respect to older adults (aged ≥ 65 years). DS-II scores were compared between older (≥ 65 years) and younger (< 65 years) adulthood and between young-old (65–74y), middle-old (75–84y), and old-old (85+y) individuals. We tested the impact of sociodemographic factors on DS-II scores within older adults.

**Results:**

The sample comprised N = 545 adults ≥ 65 years and N = 1922 adults < 65 years. DS-II scores increased in older compared to younger adults (F_(1,2465)_ = 6.1; p = 0.013; d = 0.09) and further from young-old to old-old (M_diff_ = 2.7; 95% CI 0.45, 5.46; p = 0.034). One-fourth of individuals ≥ 65 years and almost half of old-old individuals reported DS-II scores above the cut-off > 5. Living with a partner protected from demoralization in old-old individuals.

**Discussion:**

This study provides first evidence for an increased rate of demoralization in very old adults, in particular women, which is partly related to partnership status. We suggest that demoralization is considered as a crucial entity in older adulthood which can be missed by standard psychological screenings.

## Introduction

Life expectancy and thus the size of the oldest-old group (80+ years) has increased globally which is partly due to a decline in mortality (1). While suicides generally decreased from the 1980s (2) suicide rates increase with age in most countries, including Germany (3). In the year 2022, 10.119 individuals died by suicide in Germany, corresponding to a rate of 12.1 per 100.000. 75% of those were men and about one-third were at least 70 years (4). Thus, high suicide rates in older adulthood, particularly in older men, remain a major cause of death not only in Germany but worldwide (5) and therefore represent a relevant public health concern (6).

Suicide rates seem to differ among specific age sub-groups (6) so that studies have reported suicide rates in 5 to 10-year age bands throughout the whole life span. The development of suicide rates over the age groups was shown to differ between nations, showing either continuous increases with age, a bimodal pattern with a peak in middle age and oldest old, or an increase with age until middle age and then a decline or stability (3, 6, 7). However, a cross-national finding of 87 countries was that suicide rates in the 75+ years group were consistently higher than in the 65–74 years age group (3). Thus, for studies in the context of suicidality in older adulthood, it seems important to analyze different life stages, i.e. young-old (65–74 years), middle-old (75–84 years), and old-old (85+ years).

In line with the increase of depression with age across countries (8), psychiatric illnesses, in particular depression, have been identified as one of the most relevant risk factors for suicide also in older adulthood (9). However, psychiatric disorders seem to be of greater relevance for suicide in the middle-aged compared to older adults (9, 10), suggesting that a psychiatric disorder might not sufficiently explain the increasing suicide rate in the oldest old.

Several studies have investigated risk factors that are more specific to the age groups > 60 years. While the rate of completed suicides may increase with age, suicidal attempts are even more frequent in adolescents and young adults (11). Suicidal attempts significantly decreased during the life course from 200 per one suicide in teenagers to 10 per one suicide in subjects > 60 years (12) and even within older adults that died by suicide, suicidal attempts decreased from the 65–74 years group to the 75–84 years group (10). In the latter study, this was accompanied by reductions in legal and financial stressors, relationship problems, and frequency of any psychiatric problems, while in contrast, physical conditions and bereavement increased.

In a retrospective analysis of reasons for suicide in a palliative setting, physical illness turned out to be a highly relevant reason to die in older adulthood (13). Conwell et al. identified four domains of risk factors for suicide in later life: psychiatric illness, social connectedness of the older person with his or her family, friends, and community, physical illness and functional capacity (14). Moreover, Sinyor et al. performed cluster analysis in oldest-old suicide victims, showing three clusters: a) married or widowed subjects with depression and more medical health stressors, b) subjects living alone with less depression and medical health stressors and c) subjects with the highest rates of mental disorders (15). In sum, previous evidence suggests that specific sociodemographic such as social isolation, widowhood and bereavement, as well as clinical factors (i.e. dementia, cognitive impairment, and physical illness) seem to play a major role in suicide death in subjects > 65 years (11, 15, 16).

Furthermore, due to an increasing rate of cognitive and physical disabilities within the oldest old, independence and mobility are further reduced (17). Those subjects facing severe physical illnesses such as cancer are not only at higher risk for later suicide (18) but also suffer more frequently from demoralization syndrome (19, 20). Demoralization encompasses feelings of hopelessness and helplessness, a sense of incompetence or failure, and loss of meaning and purpose in life (21), which can be a distinct entity from depression (22, 23). Demoralization has been found to be independently, i.e. beyond depression severity, associated with suicidality (24, 25). In some cases a narcissistic crisis might be a mediator between physical and mental decline and suicidality in older age (26).

Supportive interventions that address demoralization have been developed in the context of patients with chronic diseases such as cancer. For instance, short-term evidence-based meaning-centered Psychotherapy typically includes assessment of the individual sources of meaning that are still present, and finding meaning through courage and commitment (27).

The relationship between demoralization and suicidality was also investigated in the light of the influential Interpersonal Theory of Suicide which posits that perceived burdensomeness and thwarted belongingness interact to foster the desire to die (28). Previous data suggested that “Meaning in life” and demoralization are constructs that mediate the relationship between perceived burdensomeness and thwarted belongingness on the one hand and suicidal ideation on the other hand (29).

An increased risk for suicide in subjects with demoralization syndrome has been confirmed in several populations, including individuals in precarious economic conditions (30), elderly women (31), college students (32), and chronic pain patients (33). However, whether demoralization is a feature of specific age groups of older adulthood remains unclear.

Demoralization is most frequently assessed by the Demoralization Scale, which is available in a refined version, the DS-II (34, 35), that was translated to German (36, 37). Yet, the DS-II has rarely been investigated in samples of the general population (37, 38). In a previous study, we investigated psychometric properties and norm values of the Demoralization Scale II (DS-II) in the German general population, indicating age-dependent DS-II scores (37). In the present manuscript, we investigated DS-II scores in older adulthood (≥ 65 years), specifically comparing the young-old, middle-old, and old-old. We hypothesize that there is not only an increase of DS-II scores with age overall but specifically in individuals ≥ 65 years and between the young-old (65–74 years), middle-old (75–84 years), and old-old (85+ years). Furthermore, as the male gender is a risk for suicide, which even increases with age, we explored gender effects on DS-II scores in the older adult cohort. Last, we explored the relationship between well-known sociodemographic risk factors for suicide (such as partnership status) and demoralization in older adulthood.

## Methods

### Data sets

Between March and May 2022, a demography consulting company, USUMA (Berlin, Germany) collected data as part of a comprehensive German household survey. A detailed description of the data collection procedure is provided elsewhere (37). In short, the country was divided into 258 regions to proportionately represent all German regions. A multistage random selection process was used to choose 6192 households (6188 valid) across these areas, aiming for a nationally representative sample. The survey involved face-to-face interviews. Of the subjects aged 16 and above, 2522 (41.2%) consented to participate. The final analysis excluded individuals younger than 18 years (44 in total), those who did not identify as male or female (n = 4), and those who were missing two or more items on one of the two subscales (n = 7), resulting in a final sample size of 2467. If there was only one missing item on any subscale, it was filled in using the mean of the valid items.

### Demoralization scale Münster

The DS-II is a simplified and shorter version of the original demoralization scale, easier to use with 16 items rated on a 3-point Likert scale (0 = never; 1 = sometimes; 2 = often). Scores vary from 0 to 32, covering two subscales: “Meaning and Purpose” (MaP) and “Distress and Coping Ability” (DaCA). Items of the DS-II and its corresponding subscale are presented elsewhere (37). The scale showed high reliability and good convergent and discriminant validity in a study with 211 palliative care patients (35). Cut-off criteria based on an extreme group design were suggested (37), distinguishing low (<25^th^ percentile; score = 0), moderate (25^th^ – 75^th^ percentile; score = 1–5), high demoralization (> 75^th^ percentile; score > 5) and very high demoralization (> 90^th^ percentile; score > 12). The scale was translated into German using rigorous methods and tested for comprehension without issues in a preliminary study (for details see (37)).

### Data analysis

One-way ANOVAs with age-group as between-subject factor (< 65y vs ≥ 65y; 65–74y vs. 75–84y vs. 85+y) were performed to test differences between older adulthood (≥ 65 years) and young to middle adulthood (< 65 years) as well as between different age-groups within older adulthood (65–74y, 75–84y, 85+y). For many analyses, approximation of normal sampling distribution was assumed due to the central limit theorem, and variance homogeneity was not violated. When comparing gender within age groups of older adulthood, sample sizes were unequal and low for the oldest age group, the assumptions of normality and variance homogeneity were not met. Thus, we decided to perform non-parametric analyses (Mann-Whitney-U-Test) to test the effects of gender. Furthermore, robust statistics for *post hoc* tests in terms of bootstrapped (n = 1000 samples, bias-corrected and accelerated) p-values and 95% confidence intervals (CI) were reported for differences between group means.

To analyze the effects of sociodemographic variables on DS-II scores, a linear regression analysis was conducted with age and gender as predictors in the first model, and partnership, income, and education that were stepwise included in the model as additional predictors.

For all analyses, critical *p* was set at 0.05. Cohen’s *d* was given as an effect size parameter for significant effects of parametric tests. Statistical analysis was carried out with IBM SPSS^®^ Statistics Software (version 28.0, IBM, Armonk, NY).

## Results

### Research sample

**Table 1** presents the sociodemographic profile of the study cohort. The group comprised 50.1% males and 49.9% females, with a mean age of *M* = 49.8 years (*SD* = 17.3; range: 18–96 years).

**Table 1:** Sociodemographic parameters of the sample.

	Total (n = 2471)		Men (n = 1237)		Women (n = 1230)	
Age, M(SD)	49.81 (17.3)		49.66 (17.3)		50.02 (17.3)	
Age groups	N	%	N	%	N	%
< 65 years	1922	77.9	967	78.2	955	77.6
≥ 65 years	545	22.1	270	21.8	275	22.4
65 – 74 years	339	62.2	173	64.1	166	60.3
75 – 84 years	173	31.7	77	28.5	96	34.9
≥ 85 years	33	6.0	20	7.4	13	4.7
Partnership (n = 545)	N	%	N	%	N	%
Living with a partner	273	51.6	175	65.5	98	37.4
Living without a partner	256	48.4	92	34.5	164	62.6
Education (n = 543)	N	%	N	%	N	%
A-level	97	17.9	66	24.5	31	11.3
Below A-level	446	82.1	203	75.5	243	88.7
Household income (n = 535)	N	%	N	%	N	%
<1250€	71	13.3	25	9.5	46	17.0
1250-<2500€	319	59.6	144	54.5	175	64.6
>2500€	145	27.1	95	36.0	50	18.5

M, mean; SD, standard deviation.

### DS-II scores in the aging population

**Figure 1:**
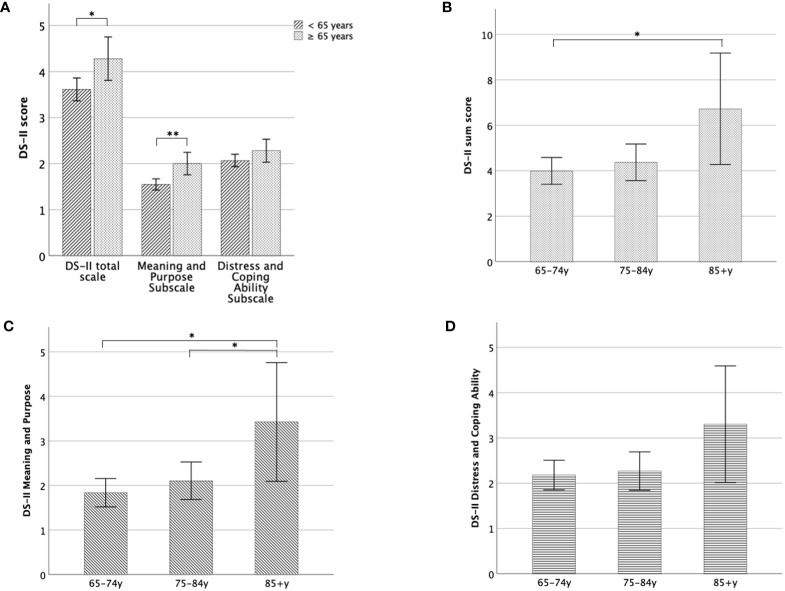
DS-II scores in older adulthood. **(A)** Mean DS-II scores of individuals < 65 years compared to those ≥ 65 years, separately for the total scale and both subscales, **(B)** Mean DS-II sum score for different age groups in the older adulthood, **(C)** Mean DS-II Meaning and Purpose subscale score for different age-groups, **(D)** Mean DS-II Distress and Coping Ability Subscale scores for different age-groups. Error bars indicate 95% CI. *significant at the 0.05-level, **significant at the 0.01-level.

Individuals ≥ 65 years showed significantly increased DS-II scores (F_(1,2465)_ = 6.1; p = 0.013; d = 0.09) and DS-II MaP scores (F_(1,2465)_ = 11.4; p < 0.001; d = 0.14) but not DS-II DaCA scores (p > 0.1); compared to subjects < 65 years (**Figure 1**).

Within older adulthood (≥ 65 years), age-group (65–74y, 75–84y, 85+y) had a moderate impact on DS-II sum scores (F_(2,542)_ = 3.6; p = 0.027; d = 0.23). This effect was even more pronounced for the DS-II MaP scores (F_(2,542)_ = 4.9; p = 0.008; d = 0.27), while differences between DS-II DaCA scores missed significance in older adulthood (p > 0.1).

As shown in **Figure 1**, the DS-II sum score was increased in old-old individuals compared to young-old (Mdiff = 2.7; 95% CI 0.45, 5.46; p = 0.034). It did not differ between young-old and middle-old (p = 1), while the difference between middle-old and old-old subjects just missed significance (p = 0.08). For the MaP scale, middle-old subjects showed similar scores as the young old (p = 0.8) whereas the old-old reported higher scores compared to both young-old (Mdiff = 1.6; 95% CI 0.44, 2.86; p = 0.007) and middle-old (Mdiff = 1.3; 95% CI 0.20, 2.50; p = 0.045).

**Figure 2:**
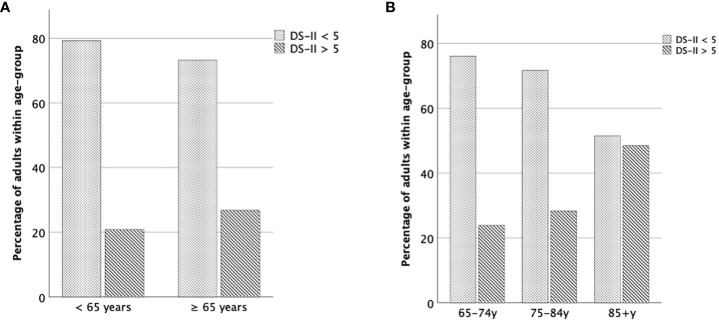
Percentage of older adults above the cut-off value. **(A)** Relative number of individuals < 65 years vs. ≥ 65 years scoring above or below cut-off (≤ 5 vs. > 5). **(B)** Relative number of individuals of each age group scoring above or below cut-off (≤ 5 vs. > 5).

The relative number of individuals scoring below or above the proposed cut-off > 5 is displayed in **Figure 2**. 20.8% of individuals aged < 65 years showed DS-II sum scores above the cut-off (> 5) whereas in older adults (≥ 65 years) 26.8% reached a DS-II score greater than 5. Within older adulthood, 23.9% of the young-old, 28.3% of the middle-old and 48.5% of the old-old scored above the cut-off (> 5).

9.5% of individuals aged < 65 years showed DS-II sum scores > 12 whereas in older adults (≥ 65 years) 10.5% reached a DS-II score greater than 12. Within older adulthood, 10.6% of the young-old, 8.7% of the middle-old and 18.2% of the old-old scored above the cutoff (> 12).

### Gender effects on demoralization in older adulthood

**Figure 3:**
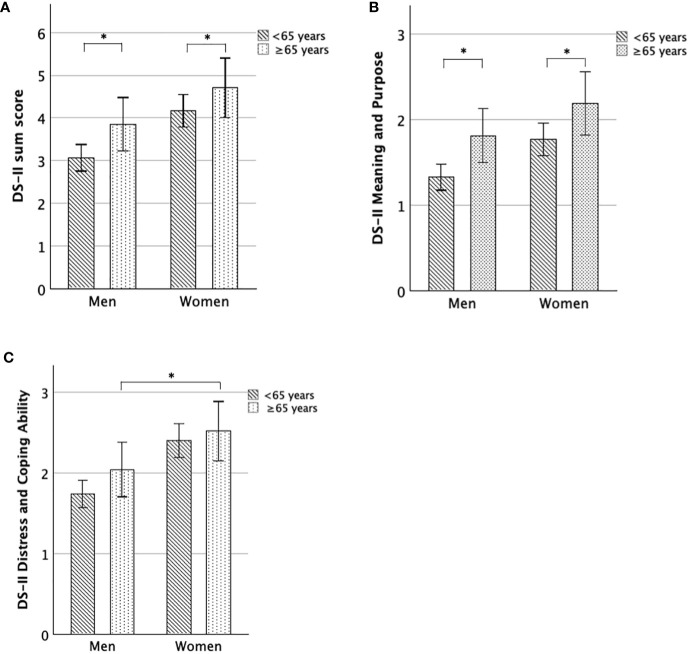
DS-II scores for women and men, comparing younger and older adulthood. **(A)** Mean DS-II sum score, **(B)** Mean DS-II Meaning and Purpose score, **(C)** Mean DS-II Distress and Coping Ability score. Error bars indicate 95% CI. *significant at the 0.05-level.

First, we tested whether DS-II scores differed between younger (< 65 years) and older (≥ 65 years) individuals and further between age groups within older adulthood, for men and women separately (**Figure 3**).

DS-II sum scores (U-Test = 116468; Z = -2.79; p = 0.005), as well as DS-II MaP scores (U-Test = 110967; Z = -4.08; p < 0.001), were significantly increased in *older men* (≥ 65 years) compared to *younger men* (< 65 years), while the differences for the DS-II DaCA scores were not significant (p = 0.08).

Similarly, DS-II MaP scores were increased in women ≥ 65 years compared to women < 65 years (U-Test = 119838; Z = -2.34; p = 0.019), DS-II sum scores were higher on a trend-level (U-Test = 121564; Z = -1.91; p = 0.056) and DS-II DaCA scores were similar between both (p > 0.2).

Within older adulthood, we found that *old-old women* (85+y) showed significantly increased DS-II sum scores (U-Test = 664; Z = -2.34; p = 0.019) and DS-II MaP scores (U-Test = 568.5; Z = -2.98; p = 0.003), but not significantly different DS-II DaCA scores (p > 0.1) compared to *young-old women* (65–74y). Moreover, *old-old women* (85+y) showed increased DS-II sum scores on a trend-level (U-Test = 420; Z = -1.93; p = 0.054) and DS-II MaP scores (U-Test = 372.5; Z = -2.42; p = 0.016), but similar DS-II DaCA scores (p > 0.2) compared to *middle-old women* (74–85y).

In contrast to the results in older women, DS-II scores (sum, MaP, DaCA) did not significantly differ between old-old and middle-old (all p > 0.24) men as well between old-old and young-old men (all p ≥ 0.07).

**Figure 4:**
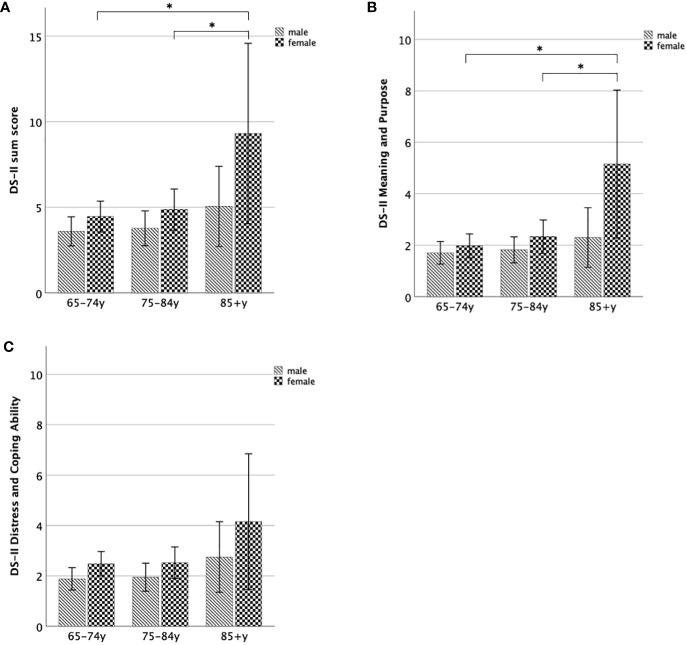
DS-II scores for women and men within subgroups of older adulthood. **(A)** Mean DS-II sum score, **(B)** Mean DS-II Meaning and Purpose score, **(C)** Mean DS-II Distress and Coping Ability score. Error bars indicate 95% CI. *significant at the 0.05-level.

In sum, both older men and older women (≥ 65 years) reported greater DS-II scores than younger adults (< 65 years), and further, within older adulthood, DS-II scores particularly increase in the oldest women (85+ years) (**Figure 4**).

Second, women ≥ 65 years showed increased DS-II DaCA scores compared to men ≥ 65 years (U-Test = 33372.5; Z = -2.12; p = 0.034), while neither DS-II sum score (p > 0.09) nor DS-II MaP scores (p > 0.5) were significantly influenced by gender in older adulthood. DS-II scores (sum, MaP, DaCA) did not significantly differ between women and men for any of the age groups of the older adulthood (all p > 0.09).

### Effects of sociodemographic factors on demoralization in the aging population

In a regression analysis within older adulthood population (age ≥ 65 years; n = 518), with age and gender as predictor variables and DS-II sum score as the dependent variable, the variables explained a significant but small part of the DS-II sum variance (R^2corr^ = 0.011; F_(2,517)_ = 3.8; p = 0.023), while only age was a significant predictor (p = 0.043). When sociodemographic variables (partnership, education, income) were stepwise included in the regression, the model again significantly improved (R^2corr^ = 0.019; F_(2,517)_ = 4.3; p = 0.005). In the final regression, partnership turned out to be the predictor explaining most of the remaining variance beyond age and gender (b_standardized_ = 0.11; p = 0.021).

Within older adulthood (65+ years), the decrease of DS-II sum scores in those living with a partner was significant (F_(1,529)_ = 11.0; p < 0.001; d = 0.29; Mdiff = -1.6; 95% CI -2.55, -0.65).

**Figure 5:**
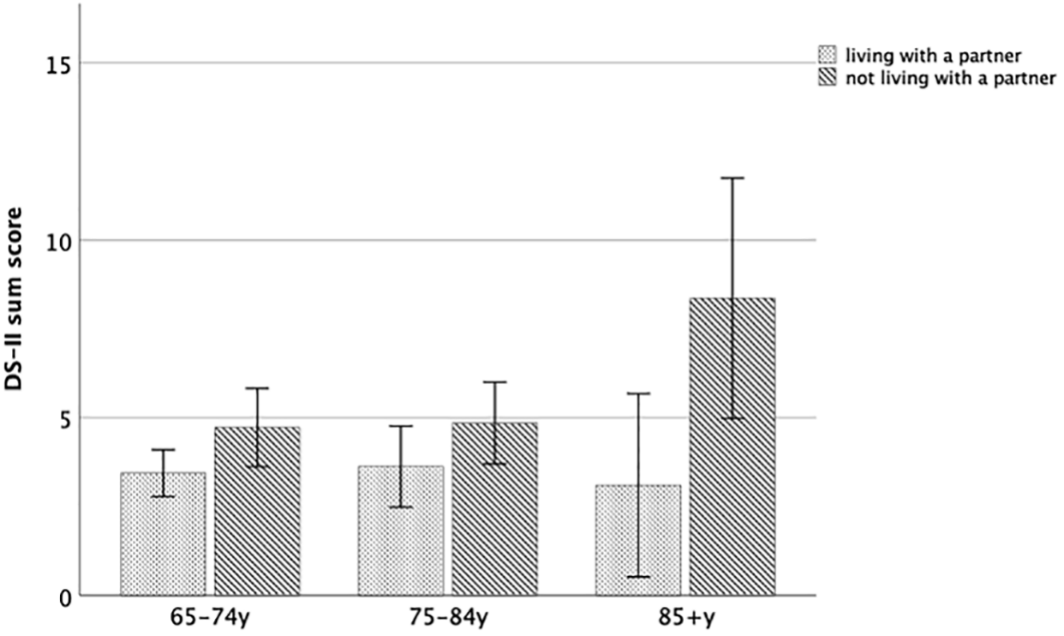
DS-II sum score with respect to partnership status within older adulthood. Error bars indicate 95% CI.

**Figure 5** shows DS-II scores with respect to whether an individual lives with a partner or not, separately for each age group within older adulthood.

Within the young-old and within the middle-old, those living with a partner did not differ in DS-II sum scores from those not living with a partner (all p > 0.14). In contrast, within old-old, those living with a partner showed DS-II sum scores that were increased on a trend level (U-Test = 63; Z = -1.92; p = 0.055).

Notably, as far more oldest men lived in a partnership (45%) than oldest women (8%), the higher rate of oldest women living without a partner (e.g. due to widowhood) parallels the increase of DS-II scores in oldest women.

## Discussion

In the current study, demoralization, which is a risk factor for suicide, was investigated in older adulthood (age ≥ 65 years) compared to younger adulthood (age < 65 years) using the DS-II in a representative sample of the German population.

This is the first study showing evidence for an increase in demoralization scores, specifically in the MaP subscale, from the younger to older adulthood and further from young-old and middle-old to old-old individuals within the general population. Furthermore, more than one-fourth of older individuals (age ≥ 65 years), and almost half of those aged 85+ years report increased demoralization (DS-II score > 5).

Most of previous empirical evidence regarding demoralization in older adulthood comes from clinical samples, in particular patients with cancer. Here, the findings for an effect of age on demoralization were mixed, showing either no association (39), a positive relationship (40) or a negative correlation (36, 41, 42) between age and demoralization, which might be influenced by treatment status (41). However, a recent meta-analysis including cancer patients with a mean age between about 50 and 68 years, and thus potentially biased by rather older participants, identified age as a risk factor for demoralization (43). This leads to the suggestion that the magnitude and directionality of an age effect on demoralization in cancer likely depend on the included age-groups. This idea is in line with our main study result that individuals ≥65 years suffer from increased demoralization when directly compared to all individuals aged <65 years which is in fact compatible with negative correlations between age and demoralization that might be observed within the range of younger adulthood, as already cautiously indicated by a previous report (37).

Yet, demoralization has rarely been studied in general populations or community-based samples. There is only one study that applied the former version of the DS-II, the DS-I, in a survey of a representative sample of the German general population. Here, in contrast to the results of the present study, subjects > 70 years did not show higher demoralization than younger age-groups (38). The reason for the inconsistency remains unclear as both studies applied similar methods. However, there is some support for the idea that the COVID-19 pandemic during which the survey was performed might account for the increase of demoralization in older adulthood in our study sample. Botto et al. (44) applied the DS-I in a sample of Italian citizens during the Italian quarantine due to COVID-19 pandemic. Indeed, they found partially high DS-I subscores, and older and female individuals, among others, had an increased risk of heightened demoralization. However, here, the literature is inconsistent. Several studies investigated psychological distress during COVID-19 pandemic, showing that older adults experienced less stress and resilient coping (45) while other studies indicated that high psychological distress also occurred in the elderly (46).

Moreover, few studies reported demoralization scores in older adulthood (including clinical samples of older adulthood), showing mean scores that were still within normal range (47, 48). However these studies did not directly compare older to younger individuals with respect to mean demoralization scores (47, 48), limiting these results’ impact on our research question.

Furthermore, we specifically found an increased MaP score in older adulthood, suggesting that older adults experience greater hopelessness, helplessness, a feeling of incompetence and a loss of purpose in life. This is in line with empirical evidence on demoralization-related constructs such as “meaning in life”, showing a small age-associated decline in purpose in life which aggravates in older adulthood (49). On the other hand, the DaCA score remained stable until older adulthood, indicating that older adults are less affected by feelings of distress and irritability and a perceived inability to cope with life. This result is in line with a wealth of literature postulating that older adults are good in transforming perspectives and goals, avoid negative stimuli, cope with stressors, thereby invest in social relationships (50–52).

The result that old-old individuals showed significantly greater DS-II sum scores than young-old, and greater DS-II MaP scores than both young-old and middle-old, is a novel finding. It supports the notion that within older adulthood, demoralization increases, reaching a peak in the old-old.

Within older adulthood, old-old women reported significantly greater DS-II scores (sum score, MaP score) than young-old and middle-old women, while this was not observed in older men. This adds to the existing literature by showing that women not only generally show higher demoralization scores than men (37, 38) but the increase of demoralization seems to occur predominantly in older women but not older men. In contrast, there is one study that investigated demoralization scores based on the DS-I specifically in a community sample of elderly women (31). They compared old women with a history of suicidality (N = 31; age 61–84 years) with old women without a history of suicidality (control group; n = 31; age 54–84 years) that were matched according to sociodemographic factors. The study found substantially increased demoralization scores in the suicidality group but a mean score within normal limits in the control group. These results however cannot directly be compared to our findings. First, our sample of the older adults was much larger (n = 545) and representative compared to the control group in that study. Second, specifically the old-old women showed the highest demoralization scores in our study, and this age group was not included in the study from Lau et al. (31).

What sociodemographic factors drive demoralization in older adulthood and particularly in older women? In the present study, we investigated whether sociodemographic factors such as income, education, or partnership had an impact on DS-II scores. Indeed, within older adulthood, partnership was the only factor beyond age and gender that remained in the final regression model, explaining about 2% of the total DS-II variance. Importantly, specifically in the oldest old (85+y), living with a partner protected from demoralization and most old-old women were not living with a partner, suggesting that the loss of partnership might explain a large part of old women’s increase of demoralization. This result is well in line with the higher life expectancy of women leading to a greater proportion of women living alone compared to men.

An increase of demoralization in older adulthood parallels findings of higher prevalence of major depression (53, 54), subclinical depression (55, 56) and completed suicides (3) in older adults. Importantly, existing literature in patients with chronic diseases indicate that demoralization can be partly distinct from depression and that demoralization is further known to be a major risk factor for suicidality independent of depression (24, 25). Notably, several studies found that the frequency of depression as the reason for suicide declines in older adulthood, suggesting that depression cannot sufficiently explain increasing suicide rates in older adulthood. Such findings in the context of increasing demoralization scores in older adulthood led us to the suggestion that demoralization could play a role. Indeed, several studies have found an association between demoralization and risk for suicide in specific populations (30–33). And widowhood has been acknowledged as a major risk factor for suicide in older adulthood (11, 15, 16) while not having a partner was also a crucial factor for demoralization in older adults in our present study. Furthermore, particularly the old-old individuals more frequently suffer from physical illnesses or just struggle with age-related normal physical changes (57) which is related to demoralization (58). Future studies should directly test whether experiencing age-related physical (and cognitive) decline might predispose to a demoralized state and whether this can explain part of the heightened risk for suicide in older adulthood.

While almost 20% of the old-old adults report demoralization syndrome (DS-II score > 12), only 0.9% of individuals 70–79 years and 0.0% of those aged 90+ years compared to 13.2% of young adults (18–29 years) receive psychotherapeutic treatment (59). Yet, evidence for the effectiveness of meaning-centered interventions in reducing demoralization is limited to patients with cancer (60, 62) but its effects on the broader target group of older adults remains unclear. As techniques of meaning-centered interventions may be implemented within few sessions and have positive effects on distress, anxiety and depression in patients with a terminal illness (27, 61) we suggest that therapists treating older adults should strongly consider incorporating such interventions into their psychotherapeutic programs. However, whether offering meaning-centered interventions to older adults can be generally recommended should be tested in future clinical trials broadly applying such techniques to older adults.

The present study has several limitations. First, while we analyzed a large sample size of older adults (N = 545), the number of included individuals aged ≥ 85 years was small (N = 33), which might limit the generalizability. Future studies could include a wider range of elderly subgroups so that effects of several psychosocial factors can be explored. A strength is that the study sample was recruited through a multistage random selection process aimed to ensure a representative sample of the German population. Second, in the present study, important related aspects such as depression, suicide ideation or behavior, and social support were not assessed so we cannot draw conclusions on the direct relationship with DS-II scores. These factors should be investigated in future studies. Third, the cross-sectional design precludes the determination of causality; future studies could adopt longitudinal designs for a better understanding of the directionality of these relationships.

In conclusion, the results of the present study indicate that demoralization is a major concern in older adults which is related to socio-environmental factors (widowhood, social isolation) and further might represent a consequence of dealing with (normal) physical changes, which is a crucial developmental task in the age-group ≥ 65 years.

Our data suggest that professionals working with older adults should consider demoralization as a complicating comorbidity more frequently occurring in the old-old. Screening for demoralization using self-report scales and adequate psychotherapeutic interventions should then be offered.

## Data availability statement

The raw data supporting the conclusions of this article will be made available by the authors, without undue reservation.

## Ethics statement

The studies involving humans were approved by Ethics committee University Leipzig, 04103 Leipzig. The studies were conducted in accordance with the local legislation and institutional requirements. The participants provided their written informed consent to participate in this study.

## Author contributions

MR: Conceptualization, Formal analysis, Methodology, Project administration, Validation, Writing – original draft, Writing – review & editing. JJ: Writing – review & editing. PL: Writing – original draft, Writing – review & editing. LJ: Writing – review & editing, Supervision. GH: Conceptualization, Formal analysis, Funding acquisition, Methodology, Project administration, Writing – original draft, Writing – review & editing. RC: Formal analysis, Methodology, Validation, Writing – review & editing.
